# Lignin-Based Nanoparticles as Both Structural and Active Elements in Self-Assembling and Self-Healing Multifunctional Hydrogels for Chronic Wound Management

**DOI:** 10.3390/pharmaceutics14122658

**Published:** 2022-11-30

**Authors:** A. Gala Morena, Sílvia Pérez-Rafael, Tzanko Tzanov

**Affiliations:** Group of Molecular and Industrial Biotechnology, Departament d’Enginyeria Química, Universitat Politècnica de Catalunya, Rambla Sant Nebridi, 22, 08222 Terrassa, Spain

**Keywords:** lignin nanoparticle, hyaluronic acid, silk fibroin, self-assembling hydrogels, chronic wounds, antibacterial, antioxidant, wound enzymes inhibition, pH-responsiveness

## Abstract

Efficient wound healing is feasible when the dressing materials simultaneously target multiple factors causing wound chronicity, such as deleterious proteolytic and oxidative enzymes and bacterial infection. Herein, entirely bio-based multifunctional self-assembled hydrogels for wound healing were developed by simply mixing two biopolymers, thiolated hyaluronic acid (HA-SH) and silk fibroin (SF), with lignin-based nanoparticles (NPs) as both structural and functional elements. Sono-enzymatic lignin modification with natural phenolic compounds results in antibacterial and antioxidant phenolated lignin nanoparticles (PLN) capable of establishing multiple interactions with both polymers. These strong and dynamic polymer-NP interactions endow the hydrogels with self-healing and shear-thinning properties, and pH-responsive NP release is triggered at neutral to alkaline pH (7–9). Despite being a physically crosslinked hydrogel, the material was stable for at least 7 days, and its mechanical and functional properties can be tuned depending on the polymer and NP concentration. Furthermore, human skin cells in contact with the nanocomposite hydrogels for 7 days showed more than 93% viability, while the viability of clinically relevant *Staphylococcus aureus* and *Pseudomonas aeruginosa* was reduced by 99.7 and 99.0%, respectively. The hydrogels inhibited up to 52% of the activity of myeloperoxidase and matrix metalloproteinases, responsible for wound chronicity, and showed a strong antioxidant effect, which are crucial features promoting wound healing.

## 1. Introduction

Polymer hydrogels are three-dimensional (3D), porous, hydrophilic networks capable of retaining large amounts of water that have received considerable attention in the biomedical field. Hydrogels can be synthesized by covalent crosslinking induced by radical polymerization, redox reactions, and enzymatic catalysis, among others [1,2], which yield non-reversible covalent bonds between the polymer chains. In physically crosslinked hydrogels, the polymeric matrix is formed by non-covalent interactions such as hydrogen bonds, ionic interactions, crystallization, metal coordination, and hydrophobic-hydrophilic interactions that usually occur at mild reaction conditions [3]. These dynamic and reversible interactions enable the hydrogels to adapt to the complex geometries of the application site and to self-heal following a stress that causes a network rupture. However, physical hydrogels are less stable and exhibit poor mechanical properties, which limits their applications. For instance, physiological conditions related to the alteration of pH or ion concentration may affect the hydrogel’s integrity. Some of the approaches used for improving the mechanical properties and durability of hydrogels include the use of nanoparticles (NPs) as reinforcing agents and the addition of a second polymer to form double-network hydrogels [4,5].

Hydrogels based either on synthetic or naturally-derived polymers have been used for biomedical applications, including tissue engineering, wound healing, sensing, and drug delivery [6,7]. Their 3D structure provides a microenvironment that promotes cell migration and proliferation [8]. Moreover, loading bioactives or cells into hydrogels increases the functionality of the materials, and properties such as antioxidant, antimicrobial, and tissue regeneration capacities are achieved [9,10]. Compared with synthetic polymers, biopolymers are preferred for biomedical applications since they are less likely to exhibit toxicity and tend to be environmentally friendly and renewable [11]. In the case of materials for wound healing, natural hydrogels such as hyaluronic acid (HA), chitosan, collagen, and silk fibroin (SF) have drawn the attention of the medical community for their physicomechanical properties, inherent biocompatibility, and biodegradability [12].

The dynamic process of wound healing takes place in three overlapping phases, i.e., hemostasis (blood clotting), inflammation (immune cell recruitment), proliferation (fibroblast growth and extracellular matrix (ECM) regeneration), and tissue remodeling (degradation of excess collagen and maturation) [13]. The healing process is regulated by many factors, including immune cells, growth factors, cytokines, and enzymes. The defective regulation of these complex events delays healing and results in the development of wound chronicity, with the healing process stalled in the inflammatory phase [14]. Chronic wounds are susceptible to infection not only by resident opportunistic bacteria normally found in the skin but also by exogenous pathogenic bacteria. All chronic wounds are heavily contaminated with microorganisms, among which *Staphylococcus aureus*, *Pseudomonas aeruginosa*, *Enterococcus faecalis*, *Staphylococcus epidermidis*, and anaerobic bacteria are the most common [15,16]. Prevention of bacterial infection has been achieved by using efficient antibacterial agents, such as topical antibiotics or silver [8]. Nevertheless, traditional antibiotics have specific targets in the bacterial cell that promote the appearance of antimicrobial resistance (AMR). Silver, in its ionic form (Ag^+^) and more recently in the form of nanoparticles (NPs), has been widely used in dressings and lotions for wound healing due to its broad range of efficacy and its action against multiple targets in the cell that would avoid resistance [17]. However, its persistence in the human body and environment raises concerns about its toxicity [18]. Research is redirecting to explore alternative antibacterial agents, including antimicrobial peptides, antibodies, polymeric NPs, and bacteriophages, among others [19]. For instance, lignin NPs have been used as antioxidant and antibacterial fillers in materials for medical applications [20,21].

In addition, non-healing wounds are also characterized by having elevated levels of reactive oxygen species (ROS), which promote biofilm formation [22] and deregulate the enzymes responsible for tissue remodeling, i.e., matrix metalloproteinases (MMPs). The elevated inflammation in chronic wounds overactivates myeloperoxidase (MPO), which produces elevated levels of hypochlorous acid. As a result, the tissue inhibitors of MMPs (TIMMPs) are degraded, and in turn, the latent form of MMPs is activated [23]. The ECM is excessively degraded when the MMPs/TIMMPs ratio is shifted towards overexpression of MMPs, which hinders the wound healing process [24]. Considering the pathophysiology of chronic wounds, an effective wound dressing should simultaneously inhibit the growth of pathogenic bacteria, reduce oxidative stress, and promote wound healing by reducing the activity of deleterious enzymes.

Previously in our group, biopolymers were combined with phenolic compounds in bulk or NP form to synthesize hydrogels for biomedical applications [1,25,26,27]. With the aim of synthesizing a metal-free, fully bio-based nanocomposite hydrogel as a potential material for wound healing, in this work, we combined two biopolymers, HA and SF, with sono-enzymatically synthesized phenolated lignin NPs (PLN) that yielded hydrogels by self-assembling. HA is a component of the ECM widely used for its high moisture retention capacity and its ability to enhance collagen deposition, re-epithelization, and vascularization, thus accelerating the wound healing process, while SF is a structural protein from *Bombyx mori* silk used for its versatility, unique mechanical properties, and tunable biodegradability. On the other hand, lignin is one of the main components of lignocellulosic biomass, and its valorization in the biomedical field is promising due to its cost-effectiveness, biocompatibility, and biodegradability. HA and SF contributed to the moisture retention capacity and structural strength, while the PLN were the gelation triggers and provided antioxidant and antibacterial properties owing to their large content of phenol groups. The rheological properties, swelling capacity, stability of the gels, and PLN release at different pHs were investigated. The potential of these materials to reduce the viability of clinically relevant Gram-negative and Gram-positive bacteria was assessed. Their ability to inhibit the enzymatic activity of MPO and MMPs was also tested. Finally, the possible cytotoxic effects of the hydrogels on human cells were studied in vitro.

## 2. Materials and Methods

### 2.1. Materials

Pharmaceutical-grade HA sodium salt from *Streptococcus equi* (MW = 600 kDa) was obtained from Lehvoss Iberia (Barcelona, Spain). N-(3- dimethylaminopropyl)-N’-ethylcarbodiimide (EDC), 2-iminothiolane hydrochloride (Traut’s reagent), adipic acid dihydrazide (ADH), 6-hydroxy-2,5,7,8-tetramethylchroman-2-carboxylic acid (Trolox), 2,4,6-trinitrobenzenesulfonic acid (TNBSA) solution at 5 *w*/*v* % in methanol were purchased from Thermo Fisher Scientific (Spain). Silk fibroin solution (5 *w*/*v* %, 100–150 kDa), Ellman’s reagent (5,5′-dithiobis-(2-nitrobenzoic acid)), 1,1-diphenyl-2-picrylhydrazyl (DPPH), phosphate buffered saline (PBS), nutrient broth (NB), Luria-Bertani (LB) with agar, Coliform ChromoSelect agar, Cetrimide agar, and Dulbecco’s Modified Eagle’s Medium (DMEM) were obtained from Sigma-Aldrich (Madrid, Spain). AlamarBlue cell viability reagent and EnzChek Gelatinase/Collagenase Assay Kit were purchased from Invitrogen, Life Technologies Corporation (Madrid, Spain). MPO from human leukocytes with an activity of 1550 U/mg solid defined as the amount of enzyme producing an increase of 1.0 absorbance unit per min at 470 nm at pH 7.0 and 25 °C, using guaiacol as a substrate, was purchased from Planta Natural Products (Vienna, Austria). Hyaluronidase with an activity of 443 U/mg solid defined as the amount of enzyme causing a change in the transmittance at 600 nm of 0.330 per min at pH 5.35 at 37 °C in a 2.0 mL reaction mixture, was purchased from Sigma-Aldrich (Spain). Bacterial strains *S. aureus* (ATCC 25923) and *P. aeruginosa* (ATCC 10145), human fibroblast cells (ATCC-CRL-4001, BJ-5ta), and human keratinocyte cells (HaCaT cell line) were obtained from the American Type Culture Collection (ATCC LGC Standards, Spain). Water was purified by the Milli-Q plus system (Millipore, Burlington, MA, USA) with 18.2 MΩ·cm resistivity before its use.

### 2.2. Modification and Characterization of HA

#### 2.2.1. Preparation of HA-ADH and HA-SH

HA was modified with ADH and Traut’s reagent in a two-step process as previously described [25] with some modifications. Briefly, HA salt (600 kDa) was dissolved in Milli-Q water (2.5 mg·mL^−1^), and ADH (45-fold molar excess) was added. After 30 min, the pH was adjusted to 4.8 with 1M HCl and EDC (a 4-fold molar excess) was added to the mixture. The pH was monitored and kept at 4.8 for 2 h by adding 1 M HCl. Afterward, the reaction was stopped by raising the pH to 7.0 with 1 M NaOH. The solution was dialyzed in water for one day using 13 kDa cut-off membranes, and then freeze-dried. For the second step, the resulting HA-ADH was dissolved in Milli-Q water (2.5 mg·mL^−1^) and Traut’s reagent, dissolved in 0.1 M pH 8 phosphate buffer, was added at a molar ratio of 1:2 (ADH:Traut’s). The reaction took place for 2 h under a nitrogen atmosphere. The modified polymer was purified by dialysis against acidified water for one day. Finally, the resulting HA-SH was lyophilized and stored at 4 °C under a nitrogen atmosphere.

#### 2.2.2. FTIR Analysis

Fourier Transform Infrared (FTIR) spectra of HA, HA-ADH, and HA-SH were recorded by a PerkinElmer Spectrum 100 FTIR spectrometer (PerkinElmer, MA, USA) in the 600−4000 cm^−1^ range, performing 64 scans for each spectrum at 4 cm^−1^ resolution. The spectrometer was equipped with an ATR accessory of germanium crystal with a high-resolution index (4.0).

#### 2.2.3. Determination of Amino and Thiol Groups

The number of primary amines in HA-ADH was assessed using the TNBSA assay. Briefly, 0.25 mL of a solution of HA-ADH was added to 0.5 mL of a 0.01 *w*/*v* % solution of TNBSA in 0.1 M of sodium bicarbonate at pH 8.5. After incubating the mixture for 2 h at 37 °C, 0.25 mL of 10 *w*/*v* % SDS and 0.125 mL of 1 N HCl were added. Then, the absorbance was measured at 335 nm. ADH standards were used to build the calibration curve and non-functionalized HA was used as a control.

The thiol content of HA-SH was determined spectrophotometrically using Ellman’s reagent. Briefly, a solution of HA-SH in 0.2 M pH 8 phosphate buffer was mixed with 0.3 mg·mL^−1^ Ellman’s reagent at a volume ratio of 1:1. After 2 h incubation in the dark, the absorbance was measured at 412 nm. The calibration curve was built using L-cysteine, and unmodified HA was used as a control.

### 2.3. Preparation and Characterization of PLN

PLN was synthesized by the enzymatically-catalyzed grafting of tannic acid onto lignin under sonication, as previously described [28]. The hydrodynamic size, polydispersity index (PDI), and ζ-potential of the particles were measured using a Zetasizer Nano Z (Malvern Instruments Inc., Worcestershire, UK). The phenolic content of PLN was determined using the Folin−Ciocalteu phenol reagent as previously described [29]. The characterization results of PLN are shown in Table S1.

### 2.4. Synthesis of HA-SH/SF Hydrogels

HA-SH was dissolved in a sodium 0.1 M pH 5.5 acetate buffer under a nitrogen atmosphere. Then, silk fibroin solution was added and stirred for 1 min. The final concentration of each polymer in the solution was 1.0 or 1.5 *w*/*v* %. PLN at different concentrations (20, 10, and 5 mg·mL^−1^) was added to the polymer mixture at a volume ratio of 20:3 (polymers:PLN). The formulations of the hydrogels are summarized in Table 1. The concentration of PLN used in the hydrogel formulations was chosen taking into account the antibacterial properties of the particles [28].

### 2.5. Rheological Characterization

The rheological characterization of the hydrogels was performed with an MCR302 rheometer (Anton Paar, Graz, Austria), equipped with electrically heated plates. The assays were carried out using a 25 mm parallel, sandblasted plate. Strain-dependent oscillatory measurements were performed at a fixed frequency (1 s^−1^) and in a range of increasing strains (from 0.1 to 10,000%). Continuous flow curves were obtained by monitoring the viscosity of the materials at increasing shear rates (0.01–100 s^−1^). The self-healing properties of the gels were studied using a 3-interval thixotropic test (3iTT) consisting of strain-dependent oscillatory measurements at a fixed frequency (1 s^−1^) with an alternating strain (between 5 and 2000%). All experiments were performed at 25 °C using a solvent trap in order to prevent dehydration during the tests.

### 2.6. Cryogenic Scanning Electron Microscopy (Cryo-SEM)

For cryo-SEM, the 1.5%_10 hydrogel and the control (a polymer mixture at 1.5% without PLN) were mounted on aluminum stubs and plunged into liquid nitrogen slush. Once the materials were frozen, they were transferred under vacuum conditions to a cryo-preparation chamber, the Quorum PP3000T (Quorum Technologies, Ltd., Lewes, UK). The preparation chamber was under high vacuum and fitted with a cold stage where the samples were cold fractured, sublimed at −90 °C for 4 min, sputter coated with platinum, and transferred to a cold stage in the chamber of the Hitachi S-3500N scanning electron microscope (Hitachi High-Tech Co., Tokyo, Japan) in the Institute of Marine Sciences of the Spanish Research Council facilities. The samples were maintained at −130 °C during the observation at an acceleration voltage of 5 kV. The average size of the pores was obtained from imaging 50 pores using ImageJ software (version 1.52a).

### 2.7. Swelling Capacity

The swelling of hydrogels was determined gravimetrically by immersing 100 mg of hydrogel in 20 mL of PBS (0.1 M, pH 7.4) at room temperature. After different incubation times, the weight of the hydrogel was determined after removing the excess water with filter paper. The swelling index was calculated as follows:

$$\mathrm{Swelling}\;\mathrm{index}\;\left(\%\right)=\left(W_{2}-W_{1}\right)/W_{1},$$

where W_1_ is the initial weight of the hydrogel prior to soaking, and W_2_ is the weight of the soaked hydrogels.

### 2.8. Stability in PBS

The hydrogel sample of 1.0%_10 was chosen to study the stability of the gels in PBS (0.1 M, pH 7.4). Samples of 300 mg of gels were immersed in 1 mL of PBS for 1, 3, and 7 days. Every 24 h, the liquid was carefully removed and replaced with fresh PBS. After the established time intervals, four samples were withdrawn from PBS and freeze-dried. The stability of the hydrogel was reported as the mean of the dry weight of the gels at each incubation time (n = 5). The statistical significance was determined using a one-way ANOVA followed by Dunnett’s multiple comparison test against time zero. *p* values less than 0.05 were considered statistically significant.

### 2.9. pH Responsiveness

The hydrogel sample of 1.0%_10 was chosen to evaluate the behavior of the gels at different pH conditions. The mechanical properties of the gels were determined by the strain-dependent oscillatory test after incubating 500 mg of hydrogel in 1 mL of 0.1 M Britton–Robinson buffer (pH 4.0, 5.0, 6.0, 7.0, 8.0, and 9.0) for 24 h. Hydrogels incubated in water were assigned as the reference. The rheological test was performed at a fixed frequency (1 s^−1^) and in a range of increasing strains (from 0.1 to 10,000%).

The release of PLN from the polymeric matrix under different pH conditions was studied by measuring the fluorescence of the liquid in which the gels were incubated. Prior to the tests, the fluorescent excitation and emission peaks of PLN were determined. The release assay consisted of immersing 60 mg of the 1.0%_10 hydrogel in 0.2 mL of 0.1 M Britton–Robinson buffer (pH 4.0, 5.0, 6.0, 7.0, 8.0, and 9.0). After different time sets, the liquid was removed, and the fluorescence was measured at λ_ex/em_ = 480/610 nm. The results are presented as the mean of three replicates ± standard deviation (SD).

### 2.10. Biodegradability and PLN Release in the Presence of Hyaluronidase

The biodegradability of the hydrogels was assessed by incubating 250 mg of the 1.0%_10 hydrogel with 1 mL of PBS (0.1 M, pH 7.4) containing 10 U·mL^−1^ of hyaluronidase for 24 h at 37 °C and 230 rpm shaking. Afterward, the liquid was carefully removed, and the tubes containing the hydrogels were freeze-dried. The control group had gels incubated only with PBS. The biodegradability of the hydrogels was reported as the mean of the dry weight of the gels (n = 4). The statistical significance was determined using a one-way ANOVA followed by Dunnett’s multiple comparison test. P values less than 0.05 were considered statistically significant.

The release of PLN in the presence of hyaluronidase was studied by measuring the fluorescence at λ_ex/em_ = 480/610 nm in the supernatant of the hydrogels (300 mg) incubated with 1 mL of PBS (0.1 M, pH 7.4) containing 10 U·mL^−1^ of hyaluronidase for 24 h at 37 °C and 230 rpm shaking. The results are presented as the mean of three replicates ± SD.

### 2.11. Antioxidant Activity

The antioxidant activity of the hydrogels was studied by measuring the decrease in absorbance of the free DPPH radical. Briefly, 30 mg of each hydrogel was incubated in 1 mL of a 100 μM DPPH solution in methanol at room temperature in the dark. At different incubation times, the supernatant was collected and the absorbance at 517 nm was measured. The assay was performed in triplicate and expressed relative to Trolox in terms of its Trolox equivalent antioxidant capacity, TEAC (μmol Trolox equiv·g^−1^ hydrogel).

### 2.12. Antibacterial Activity

The capacity of the hydrogels to inhibit bacterial proliferation was evaluated against *S. aureus* and *P. aeruginosa* by the standard flask shake method (ASTM-E2149-01) with some modifications, as previously described [25]. Briefly, 30 mg of hydrogel was immersed in a bacterial dispersion in PBS at an OD_600_ = 0.005 (corresponding to ~10^5^–10^6^ colony forming units per mL, CFU·mL^−1^) and incubated for 24 h at 37 °C and 230 rpm. The number of viable cells before and after the treatment with the gels was determined by the serial dilution method. The percent of bacterial reduction was calculated as follows:

$$\mathrm{bacterial}\;\mathrm{viability}\;\mathrm{reduction}\;\left(\%\right)=\left[\left(A-B\right)/A\right]\times 100,$$

where A and B are the average numbers of viable bacteria before and after the treatment with the hydrogels, respectively.

### 2.13. Morphology of Bacterial Cells

Morphological changes of *S. aureus* and *P. aeruginosa* treated with hydrogel were examined by scanning electron microscopy (SEM). Overnight bacterial cultures grown in NB were diluted to an OD_600_ = 0.01, and 200 μL of the suspension were treated with 60 mg of the 1.5%_10 hydrogel for 24 h at 37 °C and 230 rpm shaking. The bacterial suspension was then transferred to a 48-well plate containing silicon wafers. After 24 h at room temperature, the liquid was removed, and the bacteria remaining in the wafers were fixed overnight in a 2% paraformaldehyde and 2.5% glutaraldehyde-buffered solution. Bacteria were dehydrated by incubating the wafers with increasing concentrations of ethanol for 1 h each (25, 50, 75, and 100%). The samples were observed using a field-emission SEM (Merlin Zeiss) operating at 1 kV.

### 2.14. MPO and MMPs Inhibition

The inhibition of the MMPs’ activity in the presence of the hydrogels was studied using the Gelatinase/Collagenase Assay Kit. Briefly, 30 mg of hydrogel was incubated with 400 µL of collagenase (1.5 U·mL^−1^) for 24 h at 37 °C. After the incubation, 40 µL of gelatin substrate (125 µg·mL^−1^) was added to 100 µL of reaction, and the fluorescence was read at λ_ex_/_em_ = 493/528 nm. The percent of MMPs’ inhibition was calculated by taking the control values as 100% activity. To avoid any background caused by the presence of the hydrogels, buffer solutions containing hydrogels in the absence of collagenase were used as blanks. Controls were tubes containing only collagenase or only buffer.

The capacity of the hydrogels to inhibit the activity of MPO was studied using guaiacol as a substrate. The hydrogels (30 mg) were immersed in 200 µL of 0.1 M pH 7.5 phosphate buffer containing 48 µL of guaiacol (167 mM) and 32 µL of MPO (0.063 U·mL^−1^). After 1 h in contact with the enzyme and the substrate, the hydrogel was withdrawn, and 200 µL of the liquid was mixed with 10 µL of 1 mM H_2_O_2_ to start the reaction. Immediately after, the absorbance at 476 nm was measured every 2 min. The activity was determined by the rate of absorbance increase per min and expressed as a percentage of enzyme inhibition compared to the control (a reaction mixture with enzyme and substrate but without hydrogel). All measurements were carried out using four replicates.

### 2.15. Cytotoxicity toward Human Cells

The cytotoxicity of the hydrogels was assessed in vitro using human fibroblasts (BJ5ta cell line) and keratinocytes (HaCaT cell line). The cells were grown in DMEM supplemented with 200 mM of L-glutamine, 1% penicillin, and 10% (*v*/*v*) fetal bovine serum at 37 °C in a humidified atmosphere with 5% CO_2_. The cells were harvested at pre-confluence and seeded at a density of 62,000 cells per well on a 24-well plate containing permeable supports of tissue culture-treated polyester membrane (0.4 µm pore size). After 24 h of incubation, the cells were incubated with 30 mg of hydrogels, previously sterilized by UV, for 1 and 7 days at 37 °C. The samples and the medium were then removed, and the cell viability was assessed using 150 µL of the AlamarBlue reagent diluted in culture medium (10% *v*/*v*). After 4 h of incubation, the fluorescence was read at λ_ex/em_ = 550/590 nm. Wells containing only cells were used as the reference (growth control), while the blank was the AlamarBlue reagent incubated in the absence of cells. The percentage of cell viability was calculated as follows:

$$\mathrm{Cell}\;\mathrm{viability}\;\left(\%\right)=\left(\frac{F_{\mathrm{sample}}-F_{\mathrm{blank}}}{F_{\mathrm{growth}\;\mathrm{control}}-F_{\mathrm{blank}}}\right)\times 100,$$

the results are presented as the mean of the cell viability (%) (n = 3) ± SD.

Cell viability was further studied with fluorescence microscopy using the Live/Dead Viability/Cytotoxicity Kit (Thermo Fisher Scientific), which stains the live cells green and the dead ones red. After 7 days in contact with the hydrogels, the medium and the hydrogels were removed, and the cells were stained for 20 min with a PBS solution containing 0.1 *v*/*v* % calcein acetoxymethyl and 0.1 *v*/*v* % ethidium homodimer-1. The cells were observed using a fluorescence microscope (Nikon/Eclipse Ti-S, the Netherlands) at λ_ex/em_ = 494/517 nm for calcein acetoxymethyl and at λ_ex/em_ = 517/617 nm for ethidium homodimer-1.

### 2.16. Data Analysis

Data were analyzed using GraphPad Prism version 8.0.1 (GraphPad Software, CA, USA). Statistical significances were determined using one-way ANOVA. *p* values less than 0.05 were considered statistically significant.

## 3. Results and Discussion

### 3.1. Synthesis of Self-Assembling HA-SH/SF_PLN Hydrogels

Nano-enabled hydrogels were formed by self-assembling HA-SH, SF, and PLN. HA-SH and SF were chosen as hydrophilic polymers for the hydrogel’s matrix on account of their high solubility, molecular weight, functionality, and biocompatibility, while PLN served as gelation promoters and active agents. HA is an important component of the ECM; it is biocompatible and plays an important role in influencing cellular responses [30]. On the other hand, SF, a natural structural protein derived from the silkworm, is an FDA-approved structural protein that is safe in humans and degradable and has been used in medical devices and for synthesizing mechanically robust materials [31], which is an essential requirement for materials used in the biomedical field [32]. The crosslinking agents were lignin NPs enriched with natural phenolic compounds that have shown antioxidant and antibacterial properties and did not induce resistance in pathogenic bacterial strains [28].

In order to increase the possible polymer-NP self-assembling interactions, HA was modified with ADH and Traut’s reagent in a two-step process. The FTIR spectra of modified HA-ADH showed additional absorption at 1705 cm^−1^ (carbonyl group) and an increase in the amide I and amide II bands at 1648 cm^−1^ and 1550 cm^−1^, respectively, from the coupled hydrazide molecule (Figure S1). The absorption peaks of HA at 1406 cm^−1^ corresponding to carboxylic groups, decreased after modification with ADH, while the peak at 1376 cm^−1^ corresponding to carboxyl C=O stretching, increased. The successful modification was also corroborated by the TNBSA assay, with a primary amine content of 159 ± 52 mg ADH·g^−1^ sample. Finally, the HA-ADH was modified with Traut’s reagent to produce HA-SH with a thiol content of 12.6 ± 0.5 mg SH·g^−1^ sample.

**Figure 1:**
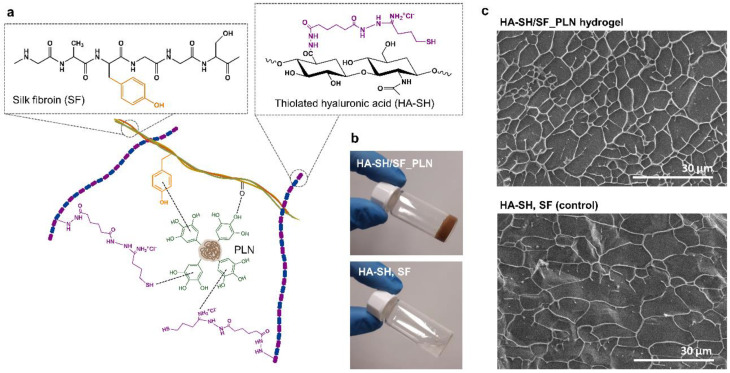
(**a**) Schematic representation of the non-covalent interactions forming the self-assembling HA-SH/SF_PLN hydrogel; (**b**) pictures of the gel obtained by mixing thiolated hyaluronic acid (HA-SH), silk fibroin (SF), and phenolated lignin nanoparticles (PLN), and the non-gelating HA-SH and SF mixture; and (**c**) cryo-SEM images of the hydrogel (HA-SH/SF_PLN) and the control without PLN (mixture of HA-SH and SF).

The hydrogels were formed in an aqueous solution under environmental conditions, and gelation of the mixture was observed immediately after mixing the polymers with PLN. Because of the presence of catechol groups in lignin, many non-covalent interactions with HA-SH and SF are possible via hydrogen bonds, π-π, and thiol-π interactions [33,34,35] forming a physically crosslinked network (Figure 1a). The strongest interactions in HA-SH are most likely between the cationic amino group from modified HA and with the phenolic groups from PLN (cation-π), whereas the strongest interaction in SF (5% tyrosine content) is most likely between the tyrosine and the phenolic group of PLN (π-π) [36]. Even if the gelation occurred within seconds, the final mechanical properties of the gels were obtained after 2 h incubation at 37 °C. This suggests that the first interactions occurring in the gels are non-covalent bonds, but during incubation, spontaneous oxidation and covalent crosslinking of phenolic groups may also occur, as well as the formation of disulfide bonds from HA-SH. Apparently, mixing HA-SH with SF did not result in a gel, which confirmed the need for PLN to form the hydrogel network (Figure 1b). Cryo-SEM images of the hydrogel revealed a microporous structure with a pore size of ~3–4 μm, while the polymer mixture in the absence of PLN (control) presented larger pores (~10 μm) (Figure 1c). The porous structure can improve the dispersibility and stability of PLN [37]. The decrease in pore size after the addition of PLN indicates a higher crosslinking degree in the hydrogel in comparison with the polymer mixture [38,39].

### 3.2. Rheological Properties of HA-SH/SF_PLN Hydrogels

**Figure 2:**
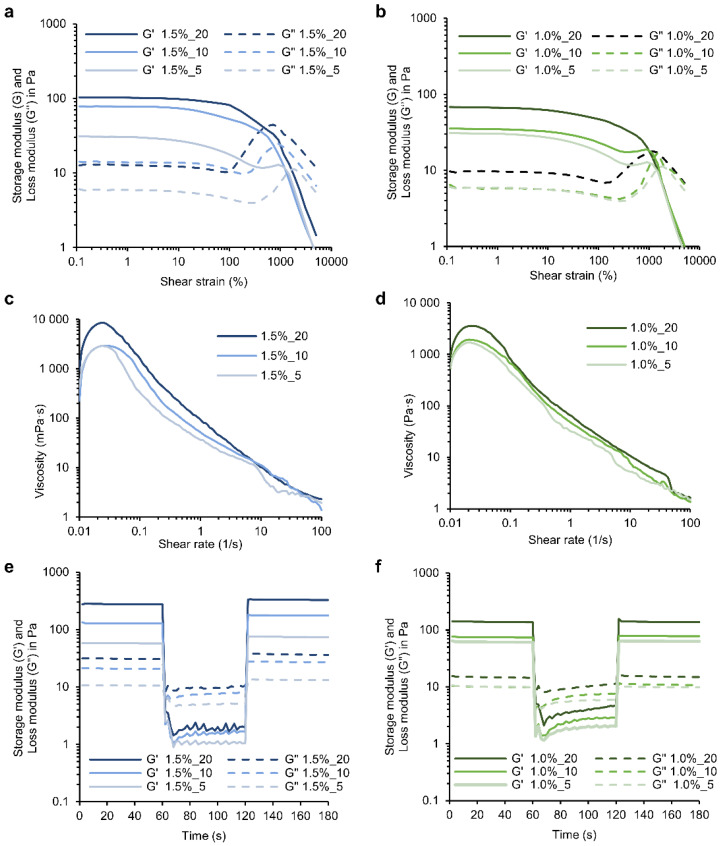
Rheological properties of the HA-SH/SF hydrogels. Strain-dependent oscillatory measurements were performed at 1 s^−1^ and increased shear strains of hydrogels prepared with (**a**) 1.5% or (**b**) 1.0% of polymers with varying PLN contents. Viscosity vs. shear rate of hydrogels prepared with (**c**) 1.5% or (**d**) 1.0% of polymers, varying PLN content. 3iTT of hydrogels prepared with (**e**) 1.5% or (**f**) 1.0% of polymers with varying PLN contents, with intervals combining 5 and 2000% strains at 1 s^−1^. All the tests were performed at 25 °C using a solvent trap. For simplification of the data interpretation, one representative sample per experimental group (n = 3) is shown.

The viscoelastic properties of the self-assembling hydrogels prepared with different concentrations of polymers and NPs were evaluated using a strain-dependent oscillatory test where the storage (G′) and loss (G″) moduli in a range of increasing strains (0.1–10,000%) were recorded (Figure 2a,b). All the tested formulations presented higher G′ values than G″, confirming their gel-like behavior. The hydrogels presented a broad linear viscoelastic region since G′ and G″ values were constant with varying deformation strains (0.1–100%). The mechanical properties of the hydrogels clearly depended on the polymers and PLN concentrations in the formulations. The gels formed with 20 and 10 mg·mL^−1^ of PLN presented G′ values of 102 and 78 Pa for hydrogels containing 1.5% HA-SH and SF, and 67 and 35 Pa in the case of 1.0% hydrogels, respectively, which are at least 2-times higher than the gels formed with the lowest concentration of PLN (5 mg·mL^−1^), suggesting a reinforced structure owing to an increased number of interactions between the polymers and the phenolic groups from PLN (Table S2) [40]. The concentration of polymers also affected the storage and loss moduli of the gels. As expected, an increase in G′ was observed at higher concentrations of HA-SH and SF, which indicated that the hydrogels with 1.5% of polymers were tougher than the ones prepared with 1.0%.

For the rheological studies, tan δ, which is the ratio of the G″ over the G′ (tan δ = G″/G′), and the flow point, which corresponds to the strain at which the sol-gel transition occurs (G′ = G″), were used as a measure of the degree of crosslinking and elasticity in the nanocomposite hydrogels (Tables S2 and S3) [40]. The flow point showed dependence on the PLN content. Increasing concentrations of PLN resulted in lower flow points, which can be related to a more structured material. Similarly, increasing the NPs concentration decreases the hydrogel elasticity, declining the tan δ values from 0.212 to 0.124 for 1.0% hydrogels and from 0.195 to 0.145 for 1.5% hydrogels. These findings highlighted the role of PLN as gelation promoters for HA-SH and SF, where NPs concentration results in a greater number of entanglements and physical nodes, enhancing the rheological properties of the nano-enabled hydrogel matrix [25].

Although mixing HA-SH and SF in the absence of PLN did not visually form a hydrogel (Figure 1b), the strain-dependent oscillatory test showed a G′ higher than G″ (Figure S2). However, G′ values were significantly lower than those obtained with the HA-SH/SF_PLN hydrogels (up to 13- and 14-fold lower for 1.0 and 1.5% formulations, respectively), the viscoelastic region was narrower, and tan δ was ~0.4–0.6. This indicated that the PLN were the primary cause of hydrogel formation, though interactions between HA, SH, and SF also contributed to the structure of the hydrogel.

The hydrogels are expected to have shear-thinning properties due to the numerous reversible interactions proposed for these nano-enabled materials, such as cation–π, hydrogen bonds, thiol–π, and π–π interactions. The viscosity of the hydrogels decreased upon increasing shear rates (Figure 2c,d), which confirmed their ability to flow on applied stress (e.g., injection through a syringe), which ensures their potential for minimally invasive delivery and conformal application. The concentration of PLN and polymers slightly affected the viscosity of the hydrogels. Concretely, increasing concentrations of PLN resulted in higher viscosity values; however, varying the concentration of polymers did not result in significant viscosity changes (Table S4).

A critical parameter for the injectability of the gels is their recovery capacity after network rupture at high strains. Step-strain measurements were then performed by combining a low strain (5%), and a high strain (2000%) that allowed the network failure (Figure 2e,f), according to previously performed strain-dependent oscillatory tests (Figure 2a,b). After applying high strains, all the hydrogels recovered their initial G′ and G″ values at low strains. This indicated that the hydrogels could recover to their initial properties after network rupture, which can be attributed to the reversible and robust nature of the non-covalently crosslinked hydrogel structure.

### 3.3. Swelling Capacity

**Figure 3:**
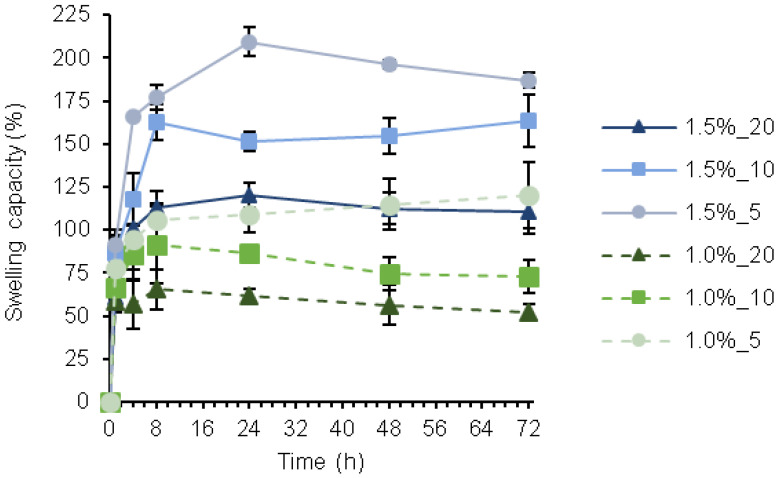
Swelling index (%) of hydrogels prepared with different concentrations of PLN and 1.5% or 1.0% of HA-SH and SF. The results are presented as mean values of swelling capacity (%) (n = 3) ± SD.

Appropriate swelling behavior of wound dressings would ensure the absorption of excessive wound exudate while maintaining moisture, which is crucial for cell growth and proliferation [8]. The swelling index of the different hydrogel formulations was studied (Figure 3) by monitoring the weight variation after immersion in PBS at pH 7.4 and room temperature. A rapid increase in the swelling was observed during the first 8 h, followed by stabilization. As expected, the swelling depended on the concentration of PLN used to prepare the gels. The formulations with less PLN (1.5%_5 and 1.0%_5) presented higher swelling, achieving values up to ~200 and ~120% for hydrogels containing 1.5 and 1.0% of biopolymers, respectively. This behavior was previously observed for hydrogels containing NPs [41] and correlates with the rheological characterization of the gels, where a higher degree of crosslinking was observed with increasing PLN content (Figure 2a,b). Indeed, a higher swelling index can be correlated with a weaker structure [42]. The amount of HA-SH and SF in the hydrogel formulation also affected the swelling index; with increasing polymer concentration and constant PLN content, a higher capacity to swell was observed. Increased swelling capacity with increasing biopolymer concentrations has previously been reported [43]. HA is a highly hydrophilic polymer capable of retaining large amounts of water and might be the main reason for the high swelling capacity of the hydrogels [41]. In short, the swelling capacity of these hydrogels can be tuned by varying the concentration of polymers (HA-SH, SF, and PLN).

### 3.4. Stability

The long-term stability of physically crosslinked hydrogels is challenging and limits their application in the medical field [32,41]. Evaluation of hydrogel stability is crucial since the physical nature of crosslinking may cause uncontrolled leaching of NPs and degradation of the polymeric matrix, which may cause toxicity. The hydrogel, at 1.0%_10, was selected as a representative example of the materials’ stability given its intermediate mechanical properties. Since the PLN are structural elements, as demonstrated by rheology, the degradation of all formulations would follow the same profile at different degradation rates depending on polymer and NP concentration. After incubating the hydrogels in PBS for 7 days, no decrease in the dry mass was observed (Table S5). A one-way ANOVA revealed that the dry mass of the hydrogels at days 1, 3, and 7 was not significantly different from the initial mass. The results demonstrated the high long-term stability of the hydrogels. Achieving high stability in self-assembling hydrogels with non-covalent interactions is challenging. In the case of HA-SH/SF/PLN hydrogels, the combination of multiple interactions, i.e., π-π, thiol-π, and hydrogen bonding, might increase the stability of the gels and reduce the possible degradation in physiological conditions. Moreover, covalent crosslinking with oxidized phenols from PLN and the polymers from the matrix may also occur, which would further increase the stability of the material.

### 3.5. Release of PLN and Hydrogel Stability in Response to pH and Hyaluronidase

The pH of healthy skin ranges from 4.2 to 6.0 [44,45]. In acute wounds, the pH oscillates during healing, often shifting from neutral to acidic with the regeneration of the epidermis. Contrarily, chronic wounds persist in an elevated alkaline environment (pH 7.2–8.9) that contributes to delayed healing [46,47]. In order to guarantee the functionality of wound dressing materials, their properties need to be maintained when exposed to different physiological changes associated with pathologies, such as elevated pH. On the other hand, the alkaline pH of chronic wounds can be used as a trigger to release active agents from dressing materials, including growth factors [48], drug-loaded polymeric NPs [49], antibiotics [50], and other drugs [51]. Controlled drug delivery systems allow the release of an active agent in response to specific internal or external stimuli, improving drug efficiency and reducing the risk of overdosing [52].

**Figure 4:**
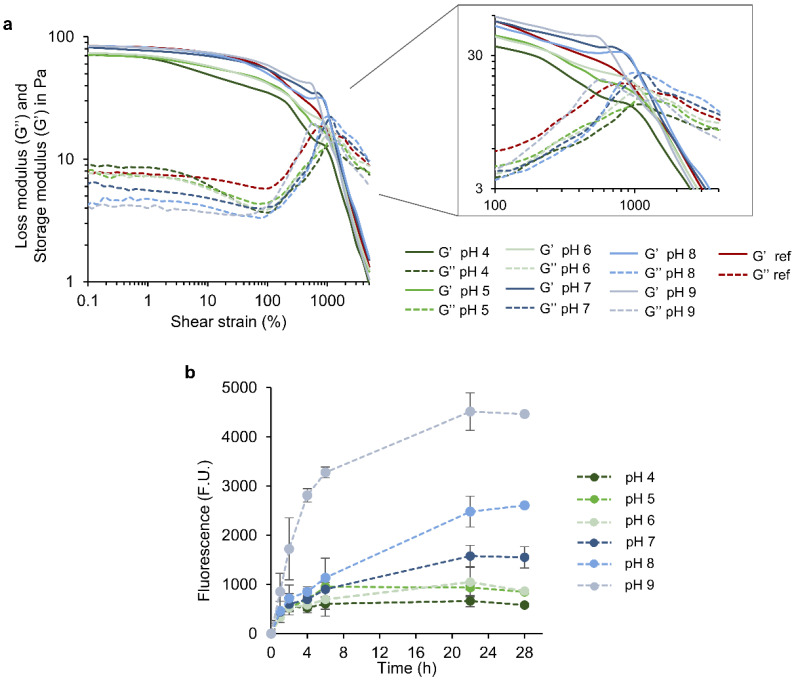
pH responsiveness of the hydrogels. (**a**) Rheological stability of the hydrogels after incubation at different pH values measured using an oscillatory test (frequency 1 s^−1^, shear strains 0.1–10,000%). For simplification, one representative sample per experimental group (n = 3) is shown. (**b**) Release of PLN from the hydrogels incubated at different pH. The results are presented as mean fluorescence values (n = 3) ± SD.

In this work, the behavior of the 1.0%_10 hydrogel at different pHs was studied in terms of rheological stability and release of PLN as active agents. Strain-dependent oscillatory tests showed that the hydrogels maintained the gel-like behavior (G′ > G″) in all the tested pH ranges (4.0–9.0) (Figure 4a). This confirms that the rheological properties of the hydrogels did not significantly vary after being exposed to acid, neutral, and alkaline conditions. The hydrogels at neutral to basic pH (7, 8, and 9) displayed lower G″ in comparison with the reference (non-treated), while G′ was maintained. Contrarily, G′ of hydrogels was lower at acid pH, while G″ decreased. Differences in the flow point were observed with increasing alkalinity, rising from 917% (reference) to 1170%. Such an increase could be correlated to a weaker structure in the hydrogel network.

The release rate and amount of PLN were clearly dependent on the pH since higher release rates with increasing pH values were observed (Figure 4b). A biphasic pattern characterized the release of PLN at alkaline and neutral pH, which consisted of an initial rapid release during the first 6 h and a slower sustained release phase. A similar profile with a significantly lower release rate resulted in an acidic pH.

The higher release rates of PLN at alkaline pH coupled with the weaker structure of the hydrogels observed by rheology indicate that polymer-NP interactions are altered, which provokes changes in the hydrogel structure. Probably, at an alkaline pH, auto-oxidation of thiol and phenol groups occurs, which weakens the polymer-NP interactions and facilitates PLN release. Moreover, the carboxylic groups from hyaluronic acid are ionized at an alkaline pH, which increases the water uptake capacity and swelling and facilitates the release of NPs [53]. Despite PLN release and the loss of some polymer-NP interactions, the hydrogels preserved their gel-like behavior. These results suggest that the hydrogels could be used as stimuli-responsive delivery materials in chronic ulcers triggered by alkaline pH. Loading the PLN with specific active substances, such as growth factors or anti-inflammatory drugs, would provide additional functionalities to the NPs related to efficient wound healing.

The PLN release and biodegradability of the hydrogels in the presence of hyaluronidase, a hydrolytic wound enzyme that degrades HA, were also studied (Table S6). The dry mass of the hydrogels after incubation with hyaluronidase did not present significant differences from that of the reference hydrogel, indicating that the hydrolytic enzyme did not degrade the hydrogel. On the other hand, higher PLN release from the hydrogels was observed in the presence of hyaluronidase in comparison with the control group (treated with PBS). Probably, hyaluronidase weakens the hydrogel structure and facilitates the release of PLN, but the presence of SF in the formulation enhances the stability of the hydrogel and delays its biodegradability [54].

### 3.6. Multiple Features of the Hydrogels for Promoting Wound Healing

The presence of pathogenic and skin bacteria contributes to the non-healing state of a wound, thus efficient management of the bacterial load is essential to progress through healing. On the other hand, chronic wounds are characterized by elevated oxidative stress and high activity of MPO and MMPs. Specific control over deleterious wound enzymes and bacterial load would enhance the healing process.

**Figure 5:**
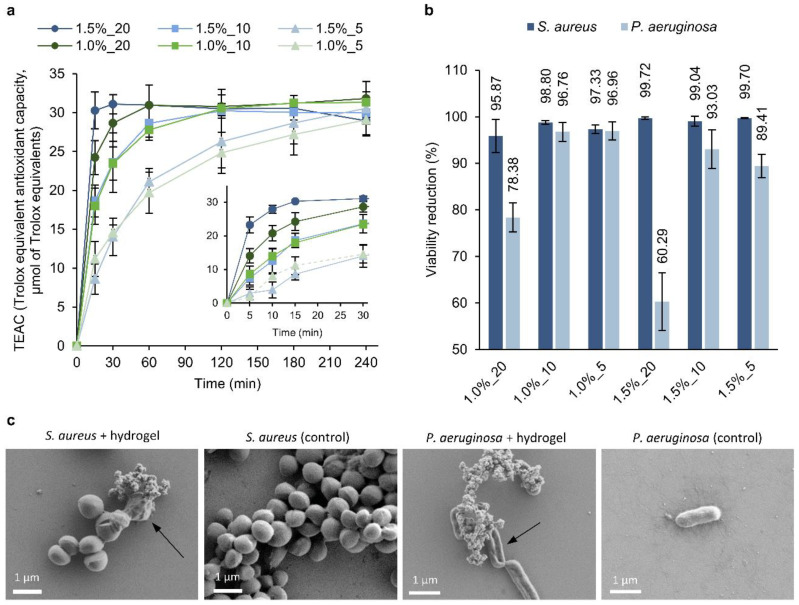
(**a**) The antioxidant activity of the hydrogels was measured using the DPPH assay. Detail of the first 30 min of assay (inset). (**b**) The antibacterial capacity of the hydrogels against *S. aureus* and *P. aeruginosa* is expressed as a percent reduction in viability (%). The results are presented as mean values of bacterial viability reduction (%) (n = 3) ± SD. (**c**) SEM images of *S. aureus* and *P. aeruginosa* treated with the hydrogels and their respective controls (without treatment).

The radical-scavenging capacity of the hydrogels was assessed using the DPPH assay. All gel formulations were able to reduce the DPPH radical, confirming their antioxidant capacity (Figure 5a). According to the results, the antioxidant activity depended on the concentration of NPs, i.e., higher PLN yielded hydrogels with a higher antioxidant capacity. The main antioxidant component of the formulations is PLN, whose numerous phenolic groups provide the modified lignin with a high antioxidant capacity [55]. Differences were also observed when varying the concentration of polymers. HA-SH could also contribute to the antioxidant capacity of the formulations [56].

The capacity of the hydrogels to reduce the viability of bacteria was assessed using two common bacteria found in wounds, the Gram-positive *S. aureus* and the Gram-negative *P. aeruginosa* (Figure 5b). The viability of *S. aureus* was reduced by 95.87–99.72%, with small differences between hydrogel formulations. The highest antibacterial formulations were those containing 1.0% of polymers, while varying the concentrations of PLN did not result in any trend. The lowest antibacterial capacity was found for a 1.5%_20 hydrogel. In general, the effect on *P. aeruginosa* was lower, achieving values ranging from 60.29 to 96.96%. The higher antibacterial activity of PLN against Gram-positive bacteria in comparison to Gram-negative bacteria was previously observed [28]. Contrary to what was expected, the gels containing the highest concentration of PLN (20 mg·mL^−1^) exhibited notably lower antibacterial capacity against *P. aeruginosa* than those prepared with 10 and 5 mg·mL^−1^. This difference can be attributed to the distinct swelling index of these hydrogels; higher swelling results in the absorption of bacteria into the hydrogel, which may contribute to the higher antibacterial effect. However, the released PLN into the medium is also expected to contribute to the antibacterial activity of the gels. The morphology of the bacterial cells treated with the hydrogels was evaluated by SEM (Figure 5c). After incubation with the hydrogels, some of the *S. aureus* cells presented irregular shapes and wrinkled surfaces that differed from the smooth and regular untreated *S. aureus*. Treated *P. aeruginosa* cells were flattened and presented depressed areas, whereas control cells were smooth and rounded. Structures formed of several particles were observed on the surface of the treated *S. aureus* and *P. aeruginosa*, which may correspond to PLN released from the hydrogels.

The advantage of using lignin as an antibacterial agent in biomedical applications is that it has unspecific and multiple antibacterial modes of action, and subsequently, the surge of AMR may be avoided [28]. In this regard, PLN are suitable antibacterial agents for controlling bacterial load and avoiding bacterial infection in wound healing materials while preventing the appearance of AMR.

**Figure 6:**
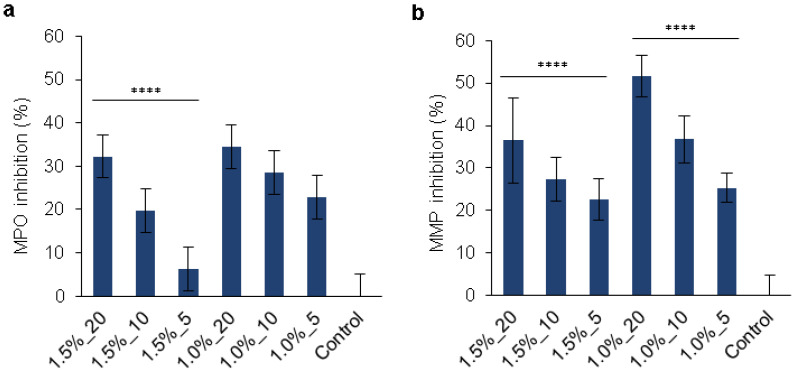
In vitro (**a**) MPO and (**b**) MMPs inhibition (%) by the hydrogels. Results are expressed in percentages of enzyme inhibition relative to the control (enzyme without hydrogel). Results are reported as mean values of enzyme inhibition (%) (n = 4) ± SD. A one-way ANOVA analysis was used to confirm the difference in MPO and MMP inhibition capacities among the different hydrogel formulations (*p* value < 0.0001 is indicated as ****).

The deregulation of enzymes and other factors in chronic wounds results in excessive proteolytic activity that provokes ECM degradation and delays healing. Thus, the control of these enzymes, i.e., MPO and MMPs, would be crucial for effective chronic wound treatment. Phenolic groups are able to act as HClO scavengers or can be directly involved in the peroxidase cycle as substrates, thereby inhibiting the chlorination activity of the enzyme [57]. Moreover, the hydrogels’ capacity to absorb fluids and proteins is also expected to diminish the activity of such enzymes in the wound bed. The capacity of the hydrogels to inhibit the MPO and MMPs’ activities was assessed in vitro (Figure 6). All the formulations except for the 1.5%_5 hydrogel were capable of significantly reducing the enzymes’ activities (by 20–52%) as a function of the amount of PLN (Table S7). The most significant MPO inhibition capacity (~34%) was achieved by the 1.5%_20 and 1.0%_20 hydrogels, independently of the amounts of HA-SH and SF employed. The MPO inhibition by the gels can be attributed to the release of PLN or the absorption of the enzyme into the negatively charged hydrogel matrix following inhibition by the thiol and phenol groups in the polymeric matrix [25,58]. A tendency was observed for MPO activity to decrease with increasing concentrations of PLN in the hydrogel’s formulation, therefore, the reduced MPO activity is probably due to the action of released PLN. The same tendency was observed for MMPs inhibition, and the highest inhibition capacity was found for the 1.0%_20 hydrogel (52% inhibition). The inhibition of MMP’s activity is most likely due to the intermolecular interaction of MMPs with the polyphenolic PLN [35,59,60].

### 3.7. Cytotoxicity Evaluation of the Hydrogels

**Figure 7:**
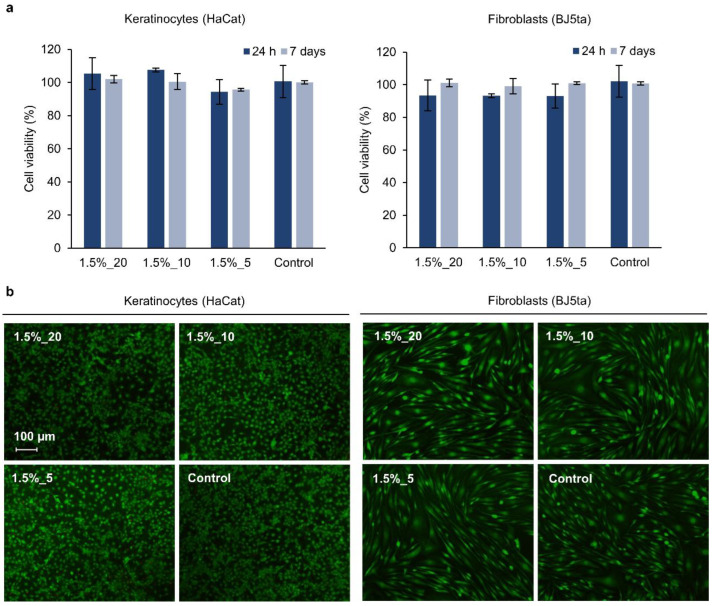
(**a**) Cell viability (%) of human keratinocytes and fibroblasts incubated with hydrogels (1.5%_20, 1.5%_10, and 1.5%_5) for 24 h assessed by the AlamarBlue assay. Results are reported as mean values of cell viability (%) ± SD (n = 3), and their statistical significance was calculated using a one-way ANOVA (*p* < 0.05). (**b**) Live/dead assay of human keratinocytes and fibroblasts treated with the hydrogels for 7 days. The assay stains the live cells green and the dead ones red.

In this study, the cell viability of human skin cells in contact with the hydrogels for 1 and 7 days was assessed. In chronic wounds, the proliferation of fibroblasts is significantly reduced in comparison with healing wounds, which deregulates tissue homeostasis and delays healing [61]. Materials used in the treatment of chronic wounds need to be biocompatible in order to minimize the loss of skin cells’ function [62]. The hydrogels did not show cytotoxicity after 1 or 7 days in contact with the cells, and the cell viability of keratinocytes and fibroblasts was no lower than 93 and 99%, respectively (Figure 7a). Statistical analysis showed no significant differences between any of the samples (*p* < 0.05). This indicated that the materials potentially released from the hydrogels did not induce cytotoxicity. From the perspective of biocompatibility, the advantage of using metal-free PLN as the only crosslinking agent in the green synthesis of hydrogels is that toxic crosslinkers and catalysts are avoided. Live/dead staining further indicated the high viability of the cells incubated with the gels, and their morphology did not suffer changes in comparison with control cells (Figure 7b). The results suggested that the application of these hydrogels for the treatment of wounds may not imply biocompatibility concerns; however, in vivo studies should be performed before clinical application.

## 4. Conclusions

We report a simple, versatile, and non-toxic route to prepare biocompatible and multifunctional nano-enabled hydrogels through the self-assembling of two biopolymers, HA-SH and SF, with the antimicrobial and antioxidant phenolated lignin NPs. Rheological studies demonstrated that PLN acted as crosslinking agents and was the primary cause of gelation. Depending on the PLN amount in the formulation, it is possible to obtain hydrogels with different rheological performances and swelling capacities to suit the final application. The non-covalent reversible polymer-NP interactions provided the hydrogels with rapid self-healing and shear-thinning properties without compromising the stability of the gels. PLN provided tunable antioxidant and antibacterial properties to the gels, which are beneficial for wound healing. The viability of clinically relevant *S. aureus* and *P. aeruginosa* was reduced by the gels up to 99.7% and 99.0%, respectively, without using antibiotics or metals. The release of active PLN was triggered in chronic wounds with alkaline pHs, while the mechanical properties of the hydrogels at this pH were not compromised. In addition, the hydrogels demonstrated the ability to inhibit the activity of deleterious wound enzymes (MPO and MMPs) as a function of PLN content, which is favorable for wound healing. Finally, the absence of cytotoxicity in fibroblasts and keratinocytes suggests that the hydrogels could be used as materials for the treatment of chronic wounds. The tunable physicomechanical and functional properties of these metal-free nanocomposites validated their potential as dressing materials to suit chronic wounds with different amounts of exudate, antibacterial load, or excessive ECM degradation.

## Figures and Tables

**Table 1:** Hydrogel formulations.

	Composition	
Hydrogel	Polymers	PLN (mg·mL^−1^)
1.5%_20	HA-SH (1.5 *w/v* %), SF (1.5 *v/v* %)	20
1.5%_10	HA-SH (1.5 *w/v* %), SF (1.5 *v/v* %)	10
1.5%_5	HA-SH (1.5 *w/v* %), SF (1.5 *v/v* %)	5
1.0%_20	HA-SH (1.0 *w/v* %), SF (1.0 *v/v* %)	20
1.0%_10	HA-SH (1.0 *w/v* %), SF (1.0 *v/v* %)	10
1.0%_5	HA-SH (1.0 *w/v* %), SF (1.0 *v/v* %)	5

## Data Availability

All data generated or analyzed during this study are included in this manuscript and its supplementary information files.

## Supplementary Materials

The following supporting information can be downloaded at: https://www.mdpi.com/article/10.3390/pharmaceutics14122658/s1. Table S1: Characterization of phenolated lignin nanoparticles (PLN): hydrodynamic size (nm), polydispersity index (PDI), ζ—potential (mv), and phenolic content (mg gallic acid equivalents, GAE per gram of sample); Table S2: Storage modulus (G′), loss modulus (G″) and damping factor (tan δ) values at 1% shear strain of different hydrogel formulations and polymer mixtures (controls); Table S3: Flow point or shear strain value (%) at which the hydrogel does not follow a gel-like behavior (G′ < G″); Table S4: Viscosity values of hydrogels at 0.1 s^−1^ and 1.0 s^−1^ shear rate; Table S5: Stability of the hydrogels in PBS at 37 °C. Dry mass (mg) of samples 1.0%_10 at time 0, 1, 3 and 7 days, and statistical significance assessed using a multiple comparison one-way ANOVA test against time 0. Results are reported as the mean of five replicates ± standard deviation (SD); Table S6: Hydrogel stability and PLN release in response to hyaluronidase. The stability was reported as dry mass (mg) of the 1.0%_10 hydrogel at time 0 and 24 h with hyaluronidase or buffer, and the statistical significance was assessed using a multiple comparison one-way ANOVA test against time 0. PLN release is reported as fluorescence units (F.U.) measured in the supernatant. All results are reported as mean values (n = 4) ± SD; Table S7: Statistical significance of the MPO and MMPs inhibition capacity of the hydrogels assessed using a multiple comparison one-way ANOVA followed by Dunnett’s post-hoc test; Figure S1: FTIR spectra of unmodified hyaluronic acid (HA), HA modified with adipic acid dihydrazide (HA-ADH) and thiolated HA (HA-SH); Figure S2: Strain-dependent oscillatory tests performed at 1 s^−1^ and 25 °C of mixtures containing a mixture of HA-SH and SF at 1.0 and 1.5% after 2 h incubation at 37 °C.
