# Supplementary material for: Lignin-Based Nanoparticles as Both Structural and Active Elements in Self-Assembling and Self-Healing Multifunctional Hydrogels for Chronic Wound Management
Source: Pharmaceutics. 2022 Nov 30;14(12):2658. doi: 10.3390/pharmaceutics14122658 (PMC9781249; doi:10.3390/pharmaceutics14122658)
Supplement: Supplementary file 1 [file pharmaceutics-14-02658-s001.zip › pharmaceutics-2030821-supplementary.pdf]

Supplementary Material

Lignin-based nanoparticles as both structural and active elements in self-assembling and self-healing multifunctional hydrogels for chronic wound management

A. Gala Morena, Sílvia Pérez-Rafael, Tzanko Tzanov*

Group of Molecular and Industrial Biotechnology, Departament d'Enginyeria Química, Universitat Politècnica de Catalunya, Rambla Sant Nebridi 22, 08222 Terrassa, Spain

*Corresponding author, email: tzanko.tzanov@upc.edu

Table S1. Characterization of phenolated lignin nanoparticles (PLN): hydrodynamic size (nm), polydispersity index (PDI), ζ –potential (mv), and phenolic content (mg gallic acid equivalents, GAE per gram of sample).

**Hydrodynamic size**	**PDI**	**ζ-potential**	**Phenolic content**
277.7 nm	0.265	–26.4 mV	325 ± 26 mg GAE·g^{-1}

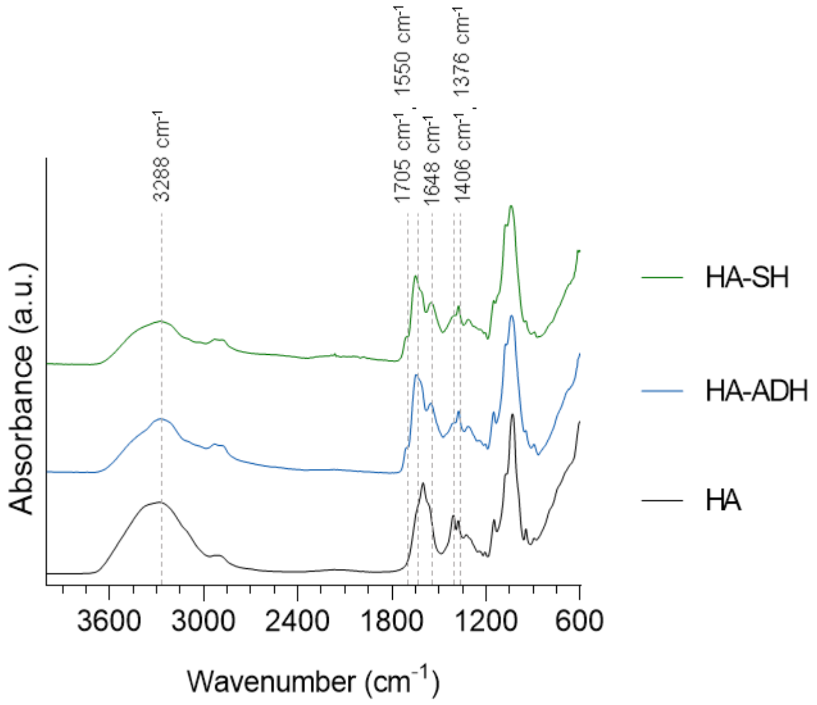

Figure S1. FTIR spectra of unmodified hyaluronic acid (HA), HA modified with adipic acid dihydrazide (HA-ADH) and thiolated HA (HA-SH).

Table S2. Storage modulus (G'), loss modulus (G'') and damping factor (tan δ) values at 1 % shear strain of different hydrogel formulations and polymer mixtures (controls).

Hydrogel sample	G' (Pa)	G" (Pa)	tan δ
HA-SH, SF, PLN_1.5%_20	102.13	12.70	0.124
HA-SH, SF, PLN _1.5%_10	77.68	13.84	0.178
HA-SH, SF, PLN _1.5%_5	59.68	12.63	0.212
Control 1.5 % (HA-SH, SF)	8.6	3.32	0.385
HA-SH, SF, PLN _1.0%_20	66.70	9.67	0.145
HA-SH, SF, PLN _1.0%_10	34.73	5.82	0.168
HA-SH, SF, PLN _1.0%_5	30.34	5.92	0.195
Control 1.0 % (HA-SH, SF)	4.65	2.05	0.442

Table S3. Flow point or shear strain value (%) at which the hydrogel does not follow a gel-like behavior (G' < G'').

Hydrogel sample	Flow point (%)
HA-SH, SF, PLN _1.5%_20	564
HA-SH, SF, PLN _1.5%_10	917
HA-SH, SF, PLN _1.5%_5	149
HA-SH, SF, PLN _1.0%_20	917
HA-SH, SF, PLN _1.0%_10	1490
HA-SH, SF, PLN _1.0%_5	1900

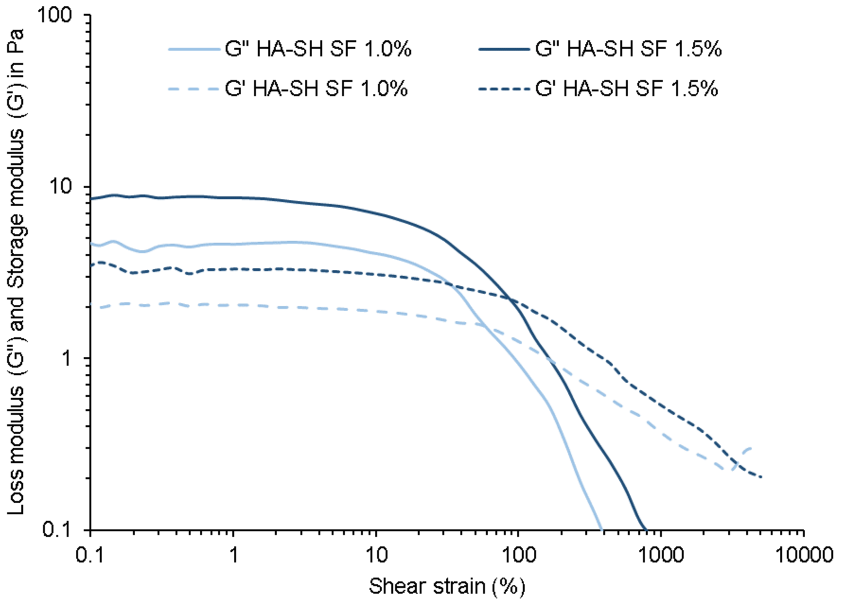

Figure S2. Strain-dependent oscillatory tests performed at 1 s^{-1} and 25 ºC of mixtures containing a mixture of HA-SH and SF at 1.0 and 1.5 % after 2 h incubation at 37 ºC.

Table S4. Viscosity values of hydrogels at 0.1 s^{-1} and 1.0 s^{-1} shear rate.

**Hydrogel sample**	**Viscosity (Pa·s) at 0.1 s^{-1}**	**Viscosity (Pa·s) at 1.0 s^{-1}**
HA-SH, SF, PLN _1.5%_20	1552.2	88.3
HA-SH, SF, PLN _1.5%_10	812.3	49.9
HA-SH, SF, PLN _1.5%_5	321.9	35.8
HA-SH, SF, PLN _1.0%_20	798.7	64.0
HA-SH, SF, PLN _1.0%_10	658.5	46.4
HA-SH, SF, PLN _1.0%_5	445.7	31.5

Table S5. Stability of the hydrogels in PBS at 37 °C. Dry mass (mg) of samples 1.0%_10 at time 0, 1, 3 and 7 days, and statistical significance assessed using a multiple comparison one-way ANOVA test against time 0. Results are reported as the mean of five replicates ± standard deviation (SD).

**Time (days)**	**0**	**1**	**3**	**7**
Dry mass (mg)	10.2 ± 0.6	11.2 ± 0.3	10.8 ± 1.0	9.8 ± 0.6
Statistical significance (One-way ANOVA)		ns[a]	ns	ns

[a]ns = not significant

Table S6. Hydrogel stability and PLN release in response to hyaluronidase. The stability was reported as dry mass (mg) of the 1.0%_10 hydrogel at time 0 and 24 h with hyaluronidase or buffer, and the statistical significance was assessed using a multiple comparison one-way ANOVA test against time 0. PLN release is reported as fluorescence units (F.U.) measured in the supernatant. All results are reported as mean values (n = 4) ± SD.

**Time (h)**	**0**	**24**	
		Hyaluronidase	Buffer
Dry mass (mg)	8.4 ± 0.1	8.0 ± 0.5	8.6 ± 0.4
Dry mass statistical significant (One-way ANOVA)		ns[a]	ns
Fluorescence (F.U.)	0	3234 ± 497	1932 ± 375

[a]ns = not significant

Table S7. Statistical significance of the MPO and MMPs inhibition capacity of the hydrogels assessed using a multiple comparison one-way ANOVA followed by Dunnett's post-hoc test.

	**Mean difference**	**95 % confidence interval**	**Significant**	**Adjusted p-value**
**MPO inhibition**				
Control vs. 1.5%_20	- 32.24	-42.97 to -21.52	Yes (****)	<0.0001
Control vs. 1.5%_10	-19.78	-29.66 to -9.906	Yes (****)	<0.0001
Control vs. 1.5%_5	-6.261	-16.14 to 3.618	No (ns)[a]	0.3662
Control vs. 1.0%_20	-34.48	-44.87 to -24.09	Yes (****)	<0.0001
Control vs. 1.0%_10	-28.48	-38.59 to -18.37	Yes (****)	<0.0001
Control vs. 1.0%_5	-22.86	-33.59 to -12.13	Yes (****)	<0.0001
**MMPs inhibition**				
Control vs. 1.5%_20	-36.46	-48.07 to -24.84	Yes (****)	<0.0001
Control vs. 1.5%_10	-27.34	-38.96 to -15.73	Yes (****)	<0.0001
Control vs. 1.5%_5	-22.66	-34.27 to -11.04	Yes (***)	0.0001
Control vs. 1.0%_20	-51.56	-63.18 to -39.95	Yes (****)	<0.0001
Control vs. 1.0%_10	-36.72	-48.33 to -25.10	Yes (****)	<0.0001
Control vs. 1.0%_5	-25.26	-36.88 to -13.64	Yes (****)	<0.0001

[a]ns = not significant
